# Crystal structure of 1-methyl-2-methyl­amino-3-nitro-1*H*-chromeno[2,3-*b*]pyridin-5(10*aH*)-one

**DOI:** 10.1107/S2056989015018241

**Published:** 2015-10-07

**Authors:** Rajamani Raja, Nataraj Poomathi, Paramasivam T. Perumal, A. SubbiahPandi

**Affiliations:** a Department of Physics, Presidency College (Autonomous), Chennai 600 005, India; b Organic Chemistry Division, CSIR Central Leather Research Institute, Adyar, Chennai 600 020, India

**Keywords:** crystal structure, chromene, hydrogen bonding, π–π stacking

## Abstract

In the title compound, C_14_H_13_N_3_O_4_, the pyran ring adopts an envelope conformation with the methine C atom as the flap. The dihedral angle between the benzene and hydro­pyridine rings is 29.33 (3)°. The methyl­amine C atom deviates from the plane of its attached ring by 0.380 (5) Å and an intra­molecular N—H⋯O hydrogen bond closes an *S*(6) ring. In the crystal, weak C—H⋯O hydrogen bonds and aromatic π–π stacking inter­actions [centroid–centroid distances vary from 3.6529 (10) to 3.6872 (10) Å] link the mol­ecules, generating a three-dimensional network.

## Related literature   

For the uses and biological importance of chromenes, see: Ercole *et al.* (2009 ▸); Geen *et al.* (1996 ▸); Khan *et al.* (2010 ▸); Raj *et al.* (2010 ▸).
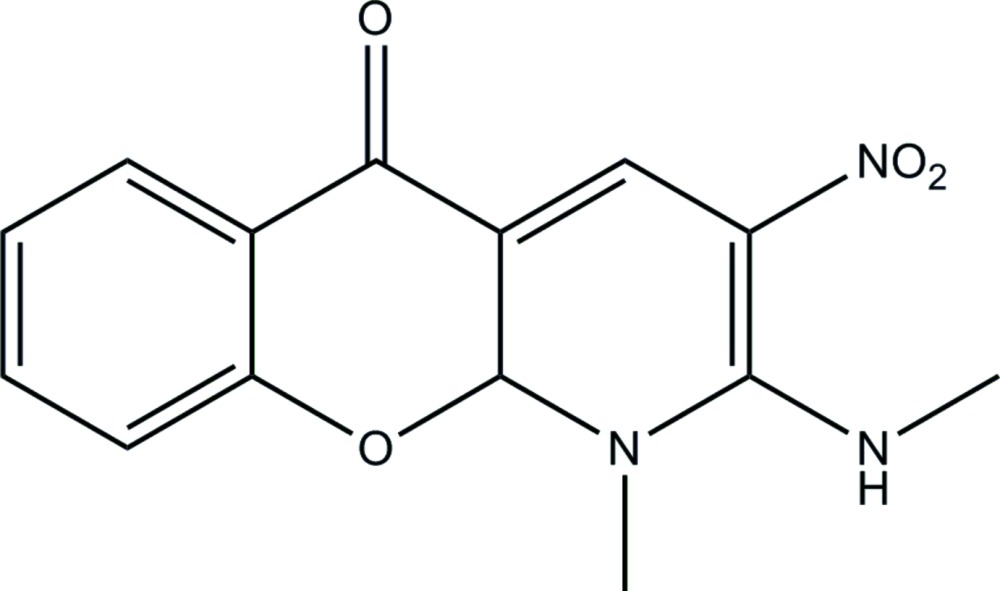


## Experimental   

### Crystal data  

C_14_H_13_N_3_O_4_
*M* *_r_* = 287.27
Orthorhombic, 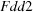

*a* = 24.0182 (13) Å
*b* = 26.8445 (14) Å
*c* = 7.9140 (4) Å
*V* = 5102.6 (5) Å^3^
*Z* = 16
Mo *K*α radiation
μ = 0.11 mm^−1^
*T* = 293 K
0.35 × 0.30 × 0.25 mm

### Data collection  

Bruker SMART APEXII CCD diffractometer
Absorption correction: multi-scan (*SADABS*; Bruker, 2008 ▸) *T*_min_ = 0.962, *T*_max_ = 0.972
12952 measured reflections
2261 independent reflections
2096 reflections with *I* > 2σ(*I*)
*R*_int_ = 0.021

### Refinement  

*R*[*F* ^2^ > 2σ(*F* ^2^)] = 0.030
*wR*(*F* ^2^) = 0.076
*S* = 1.04
2261 reflections
192 parameters
1 restraint
H atoms treated by a mixture of independent and constrained refinement
Δρ_max_ = 0.11 e Å^−3^
Δρ_min_ = −0.14 e Å^−3^

### 

Data collection: *APEX2* (Bruker, 2008 ▸); cell refinement: *SAINT* (Bruker, 2008 ▸); data reduction: *SAINT*; program(s) used to solve structure: *SHELXS97* (Sheldrick, 2008 ▸); program(s) used to refine structure: *SHELXL97* (Sheldrick, 2008 ▸); molecular graphics: *ORTEP-3 for Windows* (Farrugia, 2012 ▸); software used to prepare material for publication: *SHELXL97* and *PLATON* (Spek, 2009 ▸).

## Supplementary Material

Crystal structure: contains datablock(s) global, I. DOI: 10.1107/S2056989015018241/hb7515sup1.cif

Structure factors: contains datablock(s) I. DOI: 10.1107/S2056989015018241/hb7515Isup2.hkl

Supporting information file. DOI: 10.1107/S2056989015018241/hb7515Isup3.cml

**Fig. 1.**
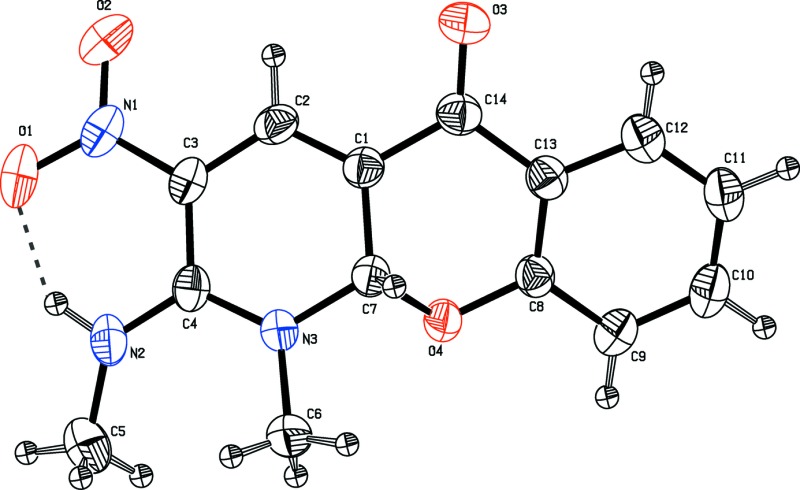
Molecular structure of the title compound, with displacement ellipsoids drawn at the 30% probability level.

. DOI: 10.1107/S2056989015018241/hb7515fig1.tif
Mol­ecular structure of the title compound, with displacement ellipsoids drawn at the 30% probability level.

**Fig. 2.**
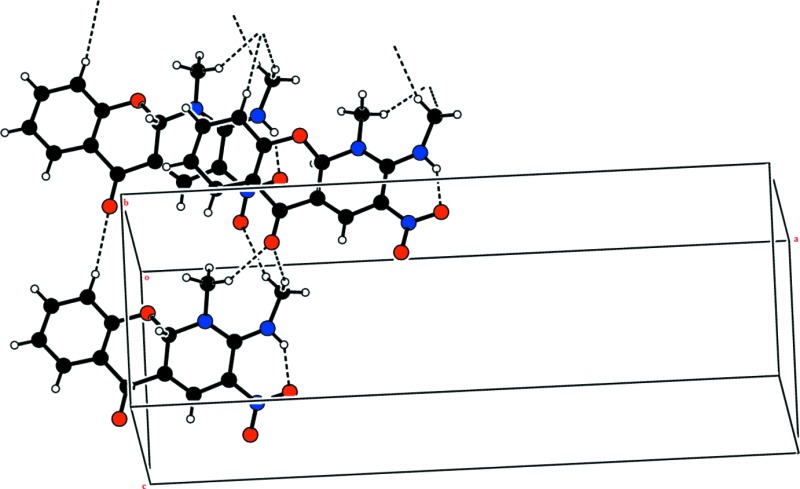
Viewed down the c axis of the crystal packing of the title compound. The hydrogen bonds are shown as dashed lines (see Table 1 for details)

. DOI: 10.1107/S2056989015018241/hb7515fig2.tif
Viewed down the c axis of the crystal packing of the title compound. The hydrogen bonds are shown as dashed lines (see Table 1 for details)

CCDC reference: 1421106

Additional supporting information:  crystallographic information; 3D view; checkCIF report

## Figures and Tables

**Table 1:** Hydrogen-bond geometry (, )

*D*H*A*	*D*H	H*A*	*D* *A*	*D*H*A*
N2H2*A*O1	0.86	1.83	2.543(2)	139
C5H5*A*O2^i^	0.96	2.46	3.376(3)	159
C5H5*C*O3^ii^	0.96	2.51	3.416(3)	157
C6H6*A*O3^ii^	0.96	2.55	3.429(2)	152
C9H9O3^i^	0.93	2.60	3.455(2)	154

Symmetry codes: (i) 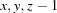; (ii) 
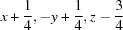
.

## supplementary crystallographic information

### S1. Comment

Chromene derivatives are very important heterocyclic compounds that have a variety of industrial, biological and chemical synthesis applications (Geen *et al.*, 1996; Ercole *et al.*, 2009). They exhibit a number of pharmacological activities such as anti-HIV, anti-inflammatory, anti-bacterial, anti-allergic, anti-cancer *etc*. (Khan *et al.*, 2010; Raj *et al.*, 2010). Against this backround, X-ray analysis of the title compound has been carried out to study its structural aspects.

The molecular structure of the title molecule is shown in Fig. 1. The pyran ring (C1—C7—O4—C8—C13—C14) adopts a envelope conformation with the deviation of atoms O4 and C14 from the mean plane through atoms (C1—C7—C8—C13) being 0.475 and −0.095 Å, respectively. The smallest displacement asymmetry parameters q_2_ and q_3_ are 0.421 (17) and −0.219 (17) Å. The ring parameters Q and phase angle θ are 0.475 (16) Å and 117.5 (2)°, respectively. The dihedral angle between the mean planes of the chromeno ring system (fusion of benzene and pyran rings) and the pyridine ring is 29.37 (7)°. The pyridine ring mean planes forms a dihedral angle of 31.22 (8)° with phenyl ring (C8—C13). The atoms O3 deviates by −0.295 Å from the chromeno ring mean plane (O4/C1—C7).

An intramolecular N—H···O and N—H···N interaction occurs. In the crystal, molecules are linked by C—H···O hydrogen bonds, forming inversion dimers with an *R*_2_^2^(8) ring motif. The molecules are linked *via* C—H···O hydrogen bonds, forming ribbons along [110] dirction. There are a number of π—π interactions present linking the ribbons and forming a three dimensional structure [*Cg*2—*Cg*3^i^ =3.6529 (10) Å, *Cg*2—*Cg*3^ii^ = 3.6871 (10) Å and *Cg*3—*Cg*2^iii^ = 3.6528 (10) Å; where *Cg*2 and *Cg*3 are the centroids of the N3/C4/C3/C2/C1/C7 and C8/C13 rings, respectively; symmetry codes: (i) −*x*,-*y*,-*z*; (ii) 1/4 + *x*,1/4 − *y*, 1/4 + *z*; (iii) −1/4 + *x*,1/4 − *y*,-1/4 + *z*].

### S2. Experimental

A mixture of 3-formylchromone (1 mmol), *N*,*N*'-dimethyl-2-nitroethene-1, 1-diamine (1 mmol) in ethanol (3 ml) and a catalytic amount (0.050 mmol) of In(OTf)_3_ was added and refluxed for about 30 minutes. The product was purified by column chromatography (5/95% Ethylacetate/petether) to afford the pure product in 94% yield. The purified compound was recrystalized from DMSO-D_6_ by using slow evaporation method to yield colourless blocks.

### S3. Refinement

N and C-bound H atoms were positioned geometrically (C–H = 0.93–0.98 Å) and allowed to ride on their parent atoms, with *U*_iso_(H) = 1.5*U*_eq_(C) for methyl H atoms and 1.2*U*_eq_(C) for all other H atoms.

### Crystal data

**Table:**

C_14_H_13_N_3_O_4_	*F*(000) = 2400
*M**_r_* = 287.27	*D*_x_ = 1.496 Mg m^−^^3^
Orthorhombic, *F**d**d*2	Mo *K*α radiation, λ = 0.71073 Å
Hall symbol: F 2 -2d	Cell parameters from 2096 reflections
*a* = 24.0182 (13) Å	θ = 2.3–25.0°
*b* = 26.8445 (14) Å	µ = 0.11 mm^−^^1^
*c* = 7.9140 (4) Å	*T* = 293 K
*V* = 5102.6 (5) Å^3^	Block, colourless
*Z* = 16	0.35 × 0.30 × 0.25 mm

### Data collection

**Table:**

Bruker SMART APEXII CCD diffractometer	2261 independent reflections
Radiation source: fine-focus sealed tube	2096 reflections with *I* > 2σ(*I*)
Graphite monochromator	*R*_int_ = 0.021
ω and φ scans	θ_max_ = 25.0°, θ_min_ = 2.3°
Absorption correction: multi-scan (*SADABS*; Bruker, 2008)	*h* = −27→28
*T*_min_ = 0.962, *T*_max_ = 0.972	*k* = −31→31
12952 measured reflections	*l* = −9→9

### Refinement

**Table:**

Refinement on *F*^2^	Primary atom site location: structure-invariant direct methods
Least-squares matrix: full	Secondary atom site location: difference Fourier map
*R*[*F*^2^ > 2σ(*F*^2^)] = 0.030	Hydrogen site location: inferred from neighbouring sites
*wR*(*F*^2^) = 0.076	H atoms treated by a mixture of independent and constrained refinement
*S* = 1.04	*w* = 1/[σ^2^(*F*_o_^2^) + (0.0468*P*)^2^ + 1.3678*P*] where *P* = (*F*_o_^2^ + 2*F*_c_^2^)/3
2261 reflections	(Δ/σ)_max_ < 0.001
192 parameters	Δρ_max_ = 0.11 e Å^−^^3^
1 restraint	Δρ_min_ = −0.14 e Å^−^^3^

### Special details

**Table:**

Geometry. All e.s.d.'s (except the e.s.d. in the dihedral angle between two l.s. planes) are estimated using the full covariance matrix. The cell e.s.d.'s are taken into account individually in the estimation of e.s.d.'s in distances, angles and torsion angles; correlations between e.s.d.'s in cell parameters are only used when they are defined by crystal symmetry. An approximate (isotropic) treatment of cell e.s.d.'s is used for estimating e.s.d.'s involving l.s. planes.
Refinement. Refinement of *F*^2^ against ALL reflections. The weighted *R*-factor *wR* and goodness of fit *S* are based on *F*^2^, conventional *R*-factors *R* are based on *F*, with *F* set to zero for negative *F*^2^. The threshold expression of *F*^2^ > σ(*F*^2^) is used only for calculating *R*-factors(gt) *etc*. and is not relevant to the choice of reflections for refinement. *R*-factors based on *F*^2^ are statistically about twice as large as those based on *F*, and *R*- factors based on ALL data will be even larger.

### Fractional atomic coordinates and isotropic or equivalent isotropic displacement parameters (Å^2^)

**Table:**

	*x*	*y*	*z*	*U*_iso_*/*U*_eq_	
C1	0.03152 (7)	0.07119 (6)	0.5103 (2)	0.0316 (4)	
C2	0.07422 (7)	0.05817 (6)	0.6093 (2)	0.0348 (4)	
H2	0.0673	0.0483	0.7200	0.042*	
C3	0.12950 (7)	0.05908 (6)	0.5492 (2)	0.0348 (4)	
C4	0.14101 (7)	0.07284 (6)	0.3758 (2)	0.0323 (4)	
C5	0.21801 (8)	0.07230 (9)	0.1521 (3)	0.0562 (6)	
H5A	0.1937	0.0565	0.0723	0.084*	
H5B	0.2531	0.0551	0.1543	0.084*	
H5C	0.2239	0.1063	0.1193	0.084*	
C6	0.10471 (8)	0.10996 (8)	0.1107 (2)	0.0514 (5)	
H6A	0.1378	0.1300	0.1095	0.077*	
H6B	0.0731	0.1305	0.0853	0.077*	
H6C	0.1078	0.0841	0.0275	0.077*	
C7	0.04068 (6)	0.08867 (6)	0.33547 (19)	0.0305 (4)	
H7	0.0273	0.1231	0.3273	0.037*	
C8	−0.04681 (7)	0.05675 (6)	0.2547 (2)	0.0315 (4)	
C9	−0.08276 (7)	0.04527 (7)	0.1229 (2)	0.0400 (4)	
H9	−0.0692	0.0389	0.0149	0.048*	
C10	−0.13915 (8)	0.04360 (7)	0.1563 (3)	0.0439 (5)	
H10	−0.1637	0.0361	0.0691	0.053*	
C11	−0.16010 (8)	0.05281 (7)	0.3162 (3)	0.0428 (5)	
H11	−0.1983	0.0528	0.3352	0.051*	
C12	−0.12390 (8)	0.06192 (7)	0.4459 (3)	0.0403 (4)	
H12	−0.1377	0.0669	0.5543	0.048*	
C13	−0.06647 (7)	0.06394 (6)	0.4184 (2)	0.0333 (4)	
C14	−0.02720 (8)	0.06748 (6)	0.5610 (2)	0.0353 (4)	
O1	0.22180 (6)	0.04252 (6)	0.6136 (2)	0.0619 (4)	
O2	0.15774 (6)	0.02632 (7)	0.79971 (18)	0.0710 (5)	
O3	−0.04181 (6)	0.06418 (6)	0.70928 (16)	0.0523 (4)	
O4	0.00911 (5)	0.05848 (4)	0.21703 (15)	0.0356 (3)	
N1	0.17083 (7)	0.04246 (6)	0.6569 (2)	0.0458 (4)	
N2	0.19283 (6)	0.07078 (5)	0.3198 (2)	0.0409 (4)	
H2A	0.2169	0.0679	0.3996	0.049*	
N3	0.09766 (5)	0.08753 (5)	0.27888 (17)	0.0339 (3)	

### Atomic displacement parameters (Å^2^)

**Table:**

	*U*^11^	*U*^22^	*U*^33^	*U*^12^	*U*^13^	*U*^23^
C1	0.0321 (9)	0.0326 (9)	0.0300 (9)	0.0013 (7)	−0.0007 (7)	−0.0038 (7)
C2	0.0399 (10)	0.0385 (9)	0.0262 (9)	−0.0023 (7)	−0.0043 (7)	−0.0041 (7)
C3	0.0317 (9)	0.0351 (9)	0.0376 (9)	0.0000 (7)	−0.0096 (8)	−0.0021 (7)
C4	0.0279 (9)	0.0299 (8)	0.0392 (9)	−0.0020 (7)	−0.0050 (8)	−0.0021 (8)
C5	0.0371 (11)	0.0692 (14)	0.0622 (13)	0.0076 (9)	0.0087 (10)	0.0000 (12)
C6	0.0379 (10)	0.0763 (14)	0.0399 (10)	0.0030 (9)	0.0018 (9)	0.0174 (10)
C7	0.0282 (9)	0.0317 (8)	0.0315 (8)	0.0012 (6)	−0.0022 (7)	−0.0017 (7)
C8	0.0280 (9)	0.0310 (9)	0.0356 (9)	0.0027 (6)	−0.0001 (7)	0.0029 (7)
C9	0.0395 (10)	0.0425 (10)	0.0378 (9)	−0.0006 (8)	−0.0079 (8)	−0.0006 (8)
C10	0.0364 (10)	0.0426 (10)	0.0527 (12)	−0.0032 (8)	−0.0143 (9)	0.0029 (9)
C11	0.0283 (10)	0.0424 (10)	0.0577 (12)	0.0009 (8)	−0.0010 (9)	0.0047 (9)
C12	0.0334 (9)	0.0393 (10)	0.0483 (11)	0.0010 (8)	0.0041 (9)	0.0015 (8)
C13	0.0318 (9)	0.0304 (8)	0.0376 (9)	0.0028 (7)	0.0014 (8)	0.0007 (7)
C14	0.0376 (10)	0.0344 (9)	0.0338 (10)	0.0029 (7)	0.0032 (8)	−0.0027 (8)
O1	0.0335 (8)	0.0824 (10)	0.0697 (11)	0.0039 (7)	−0.0197 (7)	0.0028 (9)
O2	0.0627 (10)	0.1090 (14)	0.0414 (8)	0.0052 (9)	−0.0172 (7)	0.0141 (9)
O3	0.0468 (8)	0.0771 (11)	0.0330 (7)	0.0008 (7)	0.0074 (6)	−0.0042 (7)
O4	0.0274 (7)	0.0480 (7)	0.0315 (6)	−0.0005 (5)	−0.0014 (5)	−0.0065 (5)
N1	0.0422 (10)	0.0525 (10)	0.0427 (10)	0.0006 (7)	−0.0168 (8)	−0.0015 (8)
N2	0.0252 (8)	0.0485 (9)	0.0492 (9)	−0.0011 (6)	−0.0038 (7)	−0.0018 (8)
N3	0.0263 (7)	0.0419 (8)	0.0334 (8)	0.0003 (6)	−0.0005 (6)	0.0026 (6)

### Geometric parameters (Å, º)

**Table:**

C1—C2	1.337 (2)	C7—O4	1.4527 (19)
C1—C14	1.470 (2)	C7—H7	0.9800
C1—C7	1.478 (2)	C8—O4	1.3767 (19)
C2—C3	1.410 (2)	C8—C9	1.389 (2)
C2—H2	0.9300	C8—C13	1.392 (2)
C3—N1	1.383 (2)	C9—C10	1.381 (3)
C3—C4	1.448 (3)	C9—H9	0.9300
C4—N2	1.322 (2)	C10—C11	1.384 (3)
C4—N3	1.352 (2)	C10—H10	0.9300
C5—N2	1.459 (3)	C11—C12	1.367 (3)
C5—H5A	0.9600	C11—H11	0.9300
C5—H5B	0.9600	C12—C13	1.398 (2)
C5—H5C	0.9600	C12—H12	0.9300
C6—N3	1.471 (2)	C13—C14	1.474 (2)
C6—H6A	0.9600	C14—O3	1.228 (2)
C6—H6B	0.9600	O1—N1	1.271 (2)
C6—H6C	0.9600	O2—N1	1.250 (2)
C7—N3	1.440 (2)	N2—H2A	0.8600
			
C2—C1—C14	123.98 (15)	O4—C8—C13	121.86 (15)
C2—C1—C7	121.15 (15)	C9—C8—C13	121.26 (16)
C14—C1—C7	114.82 (14)	C10—C9—C8	118.23 (18)
C1—C2—C3	121.34 (16)	C10—C9—H9	120.9
C1—C2—H2	119.3	C8—C9—H9	120.9
C3—C2—H2	119.3	C9—C10—C11	121.74 (18)
N1—C3—C2	117.53 (16)	C9—C10—H10	119.1
N1—C3—C4	121.99 (16)	C11—C10—H10	119.1
C2—C3—C4	120.25 (15)	C12—C11—C10	119.15 (17)
N2—C4—N3	123.19 (16)	C12—C11—H11	120.4
N2—C4—C3	119.11 (15)	C10—C11—H11	120.4
N3—C4—C3	117.71 (15)	C11—C12—C13	121.18 (18)
N2—C5—H5A	109.5	C11—C12—H12	119.4
N2—C5—H5B	109.5	C13—C12—H12	119.4
H5A—C5—H5B	109.5	C8—C13—C12	118.32 (16)
N2—C5—H5C	109.5	C8—C13—C14	120.29 (15)
H5A—C5—H5C	109.5	C12—C13—C14	120.99 (16)
H5B—C5—H5C	109.5	O3—C14—C1	122.65 (16)
N3—C6—H6A	109.5	O3—C14—C13	122.98 (17)
N3—C6—H6B	109.5	C1—C14—C13	114.19 (15)
H6A—C6—H6B	109.5	C8—O4—C7	112.85 (13)
N3—C6—H6C	109.5	O2—N1—O1	119.09 (16)
H6A—C6—H6C	109.5	O2—N1—C3	119.25 (16)
H6B—C6—H6C	109.5	O1—N1—C3	121.65 (16)
N3—C7—O4	106.47 (12)	C4—N2—C5	133.91 (16)
N3—C7—C1	115.21 (13)	C4—N2—H2A	113.0
O4—C7—C1	110.46 (13)	C5—N2—H2A	113.0
N3—C7—H7	108.2	C4—N3—C7	124.17 (14)
O4—C7—H7	108.2	C4—N3—C6	122.96 (15)
C1—C7—H7	108.2	C7—N3—C6	112.47 (13)
O4—C8—C9	116.82 (15)		
			
C14—C1—C2—C3	175.22 (15)	C2—C1—C14—C13	−156.47 (16)
C7—C1—C2—C3	−2.0 (3)	C7—C1—C14—C13	20.9 (2)
C1—C2—C3—N1	−176.57 (17)	C8—C13—C14—O3	−165.56 (17)
C1—C2—C3—C4	−1.9 (2)	C12—C13—C14—O3	7.0 (3)
N1—C3—C4—N2	−1.9 (2)	C8—C13—C14—C1	9.7 (2)
C2—C3—C4—N2	−176.32 (15)	C12—C13—C14—C1	−177.78 (15)
N1—C3—C4—N3	178.45 (15)	C9—C8—O4—C7	157.13 (15)
C2—C3—C4—N3	4.0 (2)	C13—C8—O4—C7	−25.6 (2)
C2—C1—C7—N3	3.6 (2)	N3—C7—O4—C8	−179.19 (12)
C14—C1—C7—N3	−173.86 (14)	C1—C7—O4—C8	55.05 (17)
C2—C1—C7—O4	124.28 (17)	C2—C3—N1—O2	1.8 (3)
C14—C1—C7—O4	−53.19 (18)	C4—C3—N1—O2	−172.78 (17)
O4—C8—C9—C10	−179.54 (16)	C2—C3—N1—O1	−179.23 (17)
C13—C8—C9—C10	3.2 (2)	C4—C3—N1—O1	6.2 (3)
C8—C9—C10—C11	−0.2 (3)	N3—C4—N2—C5	−14.2 (3)
C9—C10—C11—C12	−2.6 (3)	C3—C4—N2—C5	166.2 (2)
C10—C11—C12—C13	2.5 (3)	N2—C4—N3—C7	178.08 (15)
O4—C8—C13—C12	179.59 (16)	C3—C4—N3—C7	−2.3 (2)
C9—C8—C13—C12	−3.3 (2)	N2—C4—N3—C6	−9.7 (2)
O4—C8—C13—C14	−7.7 (2)	C3—C4—N3—C6	169.90 (16)
C9—C8—C13—C14	169.44 (16)	O4—C7—N3—C4	−124.20 (15)
C11—C12—C13—C8	0.4 (3)	C1—C7—N3—C4	−1.4 (2)
C11—C12—C13—C14	−172.27 (16)	O4—C7—N3—C6	62.89 (17)
C2—C1—C14—O3	18.8 (3)	C1—C7—N3—C6	−174.29 (15)
C7—C1—C14—O3	−163.82 (17)		

### Hydrogen-bond geometry (Å, º)

**Table:**

*D*—H···*A*	*D*—H	H···*A*	*D*···*A*	*D*—H···*A*
N2—H2*A*···O1	0.86	1.83	2.543 (2)	139
C5—H5*A*···O2^i^	0.96	2.46	3.376 (3)	159
C5—H5*C*···O3^ii^	0.96	2.51	3.416 (3)	157
C6—H6*A*···O3^ii^	0.96	2.55	3.429 (2)	152
C9—H9···O3^i^	0.93	2.60	3.455 (2)	154

Symmetry codes: (i) *x*, *y*, *z*−1; (ii) *x*+1/4, −*y*+1/4, *z*−3/4.
