# Supplementary figures and images for: Crystal structure of 1-methyl-2-methyl­amino-3-nitro-1H-chromeno[2,3-b]pyridin-5(10aH)-one
Source: Acta Crystallogr E Crystallogr Commun. 2015 Oct 7;71(Pt 11):o824–5. doi: 10.1107/S2056989015018241 (PMC4645028; doi:10.1107/S2056989015018241)

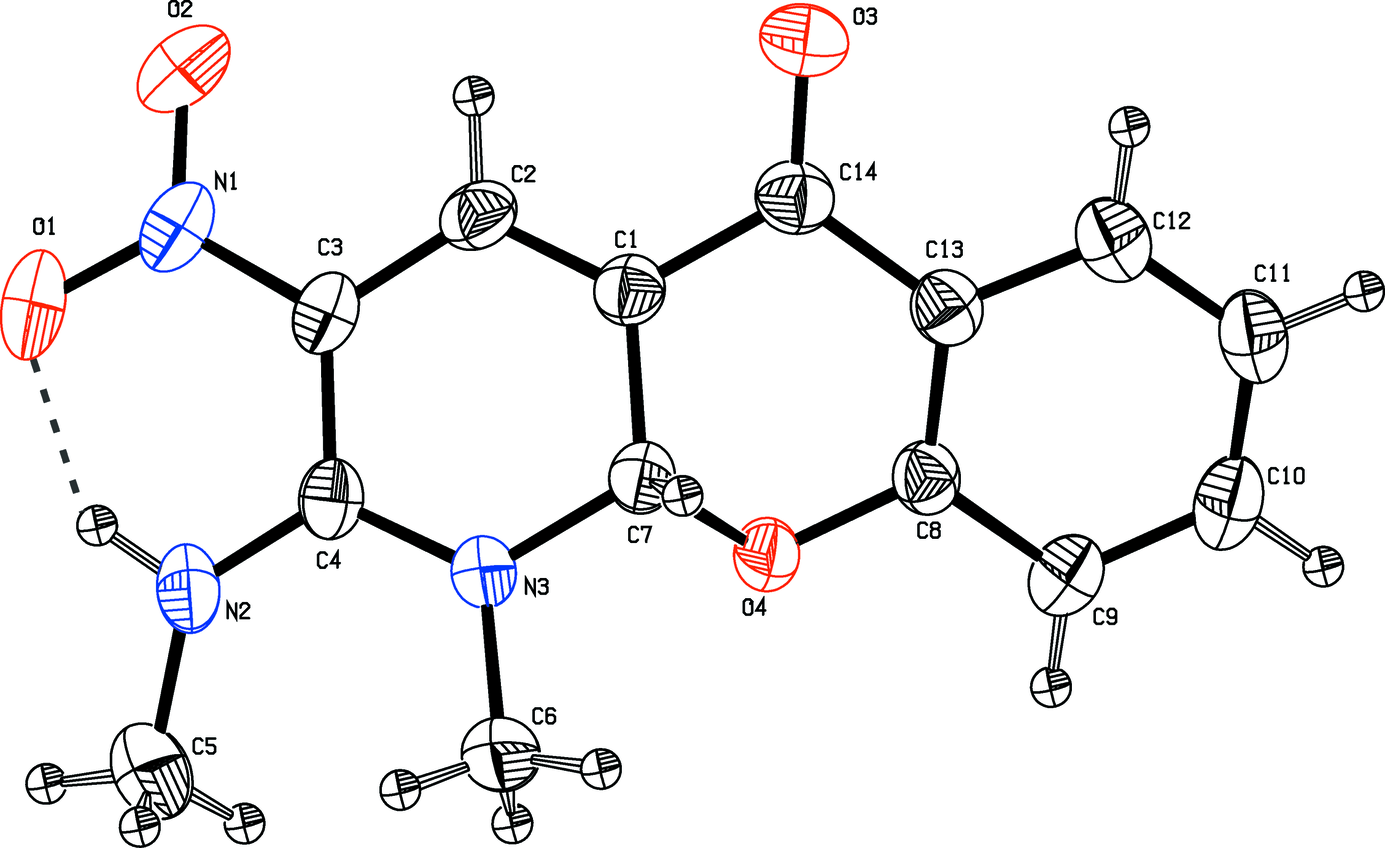

Supplement: Supplementary file 4 [file e-71-0o824-fig1.tif]

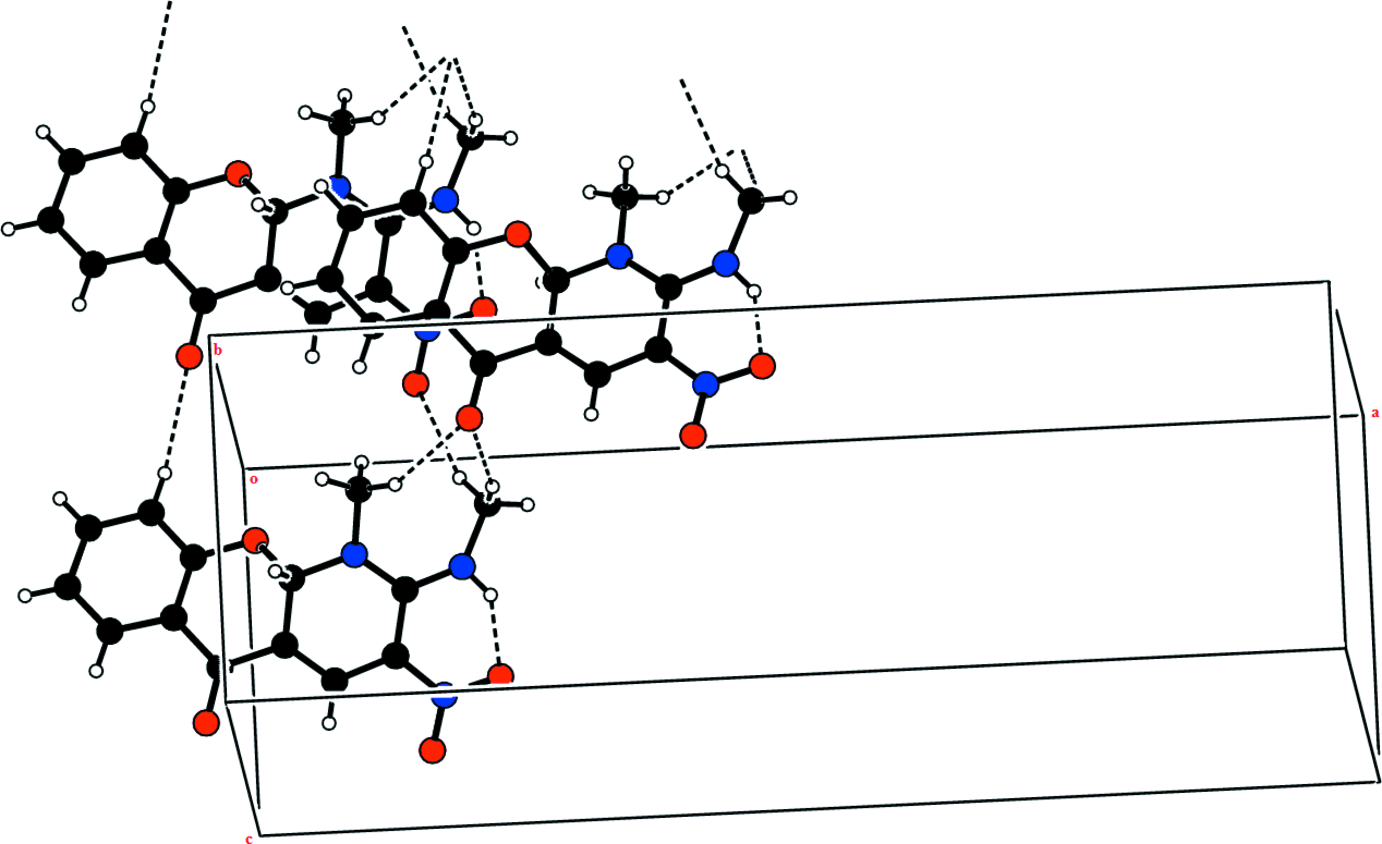

Supplement: Supplementary file 5 [file e-71-0o824-fig2.tif]
